# Trends in Demographic and Health Survey data quality: an analysis of age heaping over time in 34 countries in Sub Saharan Africa between 1987 and 2015

**DOI:** 10.1186/s13104-017-3091-x

**Published:** 2017-12-20

**Authors:** Mark Lyons-Amos, Tara Stones

**Affiliations:** School of Health Sciences and Social Work, University of Portsmouth, Portsmouth, UK

**Keywords:** Data quality, Demographic and Health Survey

## Abstract

**Objective:**

This paper evaluates one aspect of data quality within DHS surveys, the accuracy of age reporting as measured by age heaping. Other literature has explored this phenomenon, and this analysis build on previous work, expanding the analysis of the extent of age heaping across multiple countries, and across time.

**Results:**

This paper makes a comparison of the magnitude of Whipple’s index of age heaping across all Demographic and Health Surveys from 1986 to 2015 in Sub-Saharan Africa. A random slope multilevel model is used to evaluate the trend in the proportion of respondents within each survey rounding their age to the nearest age with terminal digit 0 or 5. The trend in the proportion of misreported ages has remained flat, in the region of 5% of respondents misreporting their age. We find that Nigeria and Ghana have demonstrated considerable improvements in age reporting quality, but that a number of countries have considerable increases in the proportion of age misreported, most notably Mali and Ethiopia with demonstrate increases in excess of 10% points.

## Introduction

Much attention has been paid to ensuring that basic data within Demographic and Health Surveys is correctly measured. Age heaping is frequently encountered and presents significant problems for accurate collection of data. Age heaping or age *preference* is the tendency for people to incorrectly report their age or date of birth. Individuals’ heaping behaviours favour certain ages, commonly those ending in ‘0’ or ‘5’ [1] although there is some evidence of minor heaping at eight [2].

At the most basic level, inclusion of women age 15–49 in DHS depends on accurate reports of the ages of women near the boundaries of that age interval in the survey. The inclusion of children under five (or another specified age) for the questions about child health, immunizations, and nutrition also depends on accurate reports of their birth dates. Many measures are age-specific, such as estimates of age-specific fertility rates and infant and child mortality rates [C]. Estimates of levels and trends in such rates may be affected by misreporting of ages and dates of birth for a woman and her children, or dates of death for her children. Age displacement of children can seriously distort estimates of current levels and recent trends in fertility and mortality and is by no means unique to DHS surveys: evaluation of censuses and community surveys have revealed severe age misreporting [2–4]. Additionally, age heaping can have implications for the quality of analyses into other phenomena, such as cause specific death rates [5]. This has led to a plethora of studies evaluating the quality of basic demographic data in the DHS in a variety of contexts [6–9].

Our analysis provides an evaluation of how the excess proportion varies over time and between countries. This analysis expands on previous works [7, 10], increasing the range of countries evaluated as well as capturing trends across time, to account for potential structural change which may improve the quality of retrospective data [8] as well as better data collection techniques [11, 12]. Our working hypothesis is that there should be a falling trend in the proportion of ages showing digit preference across time.

As such, this paper addresses two major research aims:

1. Capturing the overall trend in the quality of age recall data across multiple waves of DHS surveys.
2. Evaluate the extent of cross national variation in the extent of age heaping.

## Main text

### Method

#### Data

DHS are nationally representative, cross-sectional household surveys with multi-stage cluster sampling designs. Respondents are women of reproductive age (which are defined by DHS as between 15 and 49 years) and only women between these ages are interviewed. While a male dataset is available, and digit preference is also exhibited albeit to a lower extent for males [4], collection is much less consistent (especially for early surveys) and so the analysis is limited to females only. Exact details of the sampling designs are available on a country by country basis, and data sets can be downloaded on request from the provider. We restrict our analysis to the Sub Saharan Africa region to minimize the extent to which cross cultural variation in age heaping may play a role [13].

#### Whipple’s index of age heaping

This analysis uses Whipple’s index of age heaping to measure age data quality [4, 13]. Whipple’s index measures the excess proportion of ages ending in either 0 or 5. Where no ages are heaped, we expect this index to take the value 0.2. Deviation from this number indicates some degree of terminal digit preference, for example 0.25 indicating that 5% of ages have been heaped at either a zero or five terminal digit.

#### Regression model

We specify the dependent variable in our model as the excess proportion of ages ending in 0 or 5 from (Whipple’s index of heaping), denoted as *y*_*tj*_ where *y* is the proportion of respondents with heaped ages, indexed by year of survey *t* and country *j*. Survey years are hierarchically nested within countries. We specify a multilevel model in the form of Eq. 1, where the logit of the index of heaping is a function of the year of the survey with intercountry variation captured a random effect parameter at the country level, *ν*_*j*._

1 $$ \begin{aligned} {\text{logit}}\left( {y_{tj} } \right) = \beta_{0} + \beta_{1} t + \nu_{0j} \hfill \\ \nu_{0j} \sim N\left( {0, \;\sigma^{ 2} } \right) \hfill \\ \end{aligned} $$

To overcome the non-linearity of the proportion of age heaped at zero, we use a logit link to allow the specification of the model in the linear form of Eq. 1. We explored different specifications of the year of survey parameter by introducing square and cubic terms for the effect of year to account for non-linearity but neither of these specifications improved model fit on -2LogLikelihood significance tests.

We performed tests for differences in the trend in the proportion of ages heaped over time by introducing a random slope parameter at the country level. This model is described in Eq. 2

2 $$ \begin{aligned} {\text{logit}}\left( {y_{tj} } \right) = \beta_{0} + \beta_{1} t + \upsilon_{0j} + \upsilon_{1j} t \hfill \\ \nu_{0j} \sim N\left( {0,\;\sigma^{2} } \right), \;\nu_{1j} \sim N(0,\;\sigma^{2} ). \hfill \\ \end{aligned} $$

In Eq. 2, the random effect parameter *ν*_1*j*_ allows deviation from the overall trend in Whipple’s index of heaping over time according to indexation by country *j*. This parameter is allowed to correlate with *ν*_0*j*_.

Model estimation is conducted by taking the logit of Whipple’s index of heaping, and using this as the response variable in a linear multilevel analysis. Models are estimated using MlwiN 2.36 [14], with Restricted Iterative Generalised Least Square (2nd order Penalised Quasi Likelihood) estimation used to account for the low number of observations per country.

### Results

The countries included, the years of survey and the proportion of 0 and 5 terminal digits are presented in Table 1. The overwhelming majority of surveys exhibit proportions.

**Table 1:** Countries for analysis and years of survey with proportion of ages with terminal digit 0 or 5

Country	Year of survey																													
	1986	1987	1988	1989	1990	1991	1992	1993	1994	1995	1996	1997	1998	1999	2000	2001	2002	2003	2004	2005	2006	2007	2008	2009	2010	2011	2012	2013	2014	2015
Benin											0.23					0.32					0.36					0.34				
Burkina Faso								0.25						0.27				0.27							0.26					
Burundi		0.23																							0.26					
Cameroon						0.26							0.26						0.26							0.26				
Chad												0.36							0.34											0.41
Comoros											0.26																0.28			
Congo																				0.23							0.21			
Cote dIvoire									0.26					0.25													0.23			
DRC																						0.23							0.23	
Ethiopia															0.30					0.37						0.35				
Gabon															0.23												0.20			
Gambia																												0.28		
Ghana			0.31					0.28					0.28					0.25					0.27						0.25	
Guinea														0.32						0.36							0.33			
Kenya				0.27				0.24					0.24					0.23					0.25						0.24	
Lesotho																			0.21					0.22					0.21	
Liberia	0.27																					0.24						0.24		
Madagascar							0.24					0.24							0.23					0.25						
Malawi							0.23								0.25				0.24						0.23					
Mali		0.23									0.31					0.31					0.34							0.34		
Mozambique												0.24						0.23								0.23				
Namibia							0.22								0.22							0.20						0.21		
Niger							0.36						0.33								0.37						0.37			
Nigeria					0.44													0.35					0.36					0.35		
Rwanda							0.23								0.24					0.22			0.23		0.22					0.22
Sao Tome and Principe																								0.20						
Senegal	0.23						0.28					0.23								0.27						0.29	0.27		0.26	
Sierra Leone																							0.37					0.34		
Swaziland																					0.21									
Tanzania							0.25				0.24			0.26						0.25					0.25					
Togo			0.27										0.28																0.29	
Uganda				0.25						0.26						0.26					0.24					0.25				
Zambia							0.20				0.20					0.21						0.22							0.22	
Zimbabwe			0.23						0.23					0.22							0.21					0.22				

Figure within table indicate proportion of responses ending in 0 or 5

Values within table above 0.20 indicate digit preference for 0 or 5

Empty spaces within table indicate survey not carried out in that year

Results from the modelling are presented in Table 2. We find no evidence of a trend toward an improvement in the proportion of ages heaped, with the coefficient from both Model I and Model II being both statistically non-significant and substantively small.

**Table 2:** Estimated multilevel model for proportion of ages heaped

	Model I: random intercept model		Model II: random slope model	
	Parameter estimate	95% confidence interval	Parameter estimate	95% confidence interval
Fixed effect parameters				
Survey year (centred)	0.004	(− 0.004, 0.012)	0.003	(− 0.010, 0.015)
Intercept	− 3.032		− 3.025	
Random effect parameters				
Random intercept *ν*_0*j*_	0.836	(0.416,1.256)	0.786	(0.390,1.182)
Random slope *ν*_1*j*_	–	–	0.001	(0.000, 0.001)
Intercept-slope covariance	–	–	0.004	(− 0.007, 0.015)

Model based on 2nd order PQL RIGLS

**Fig. 1:**
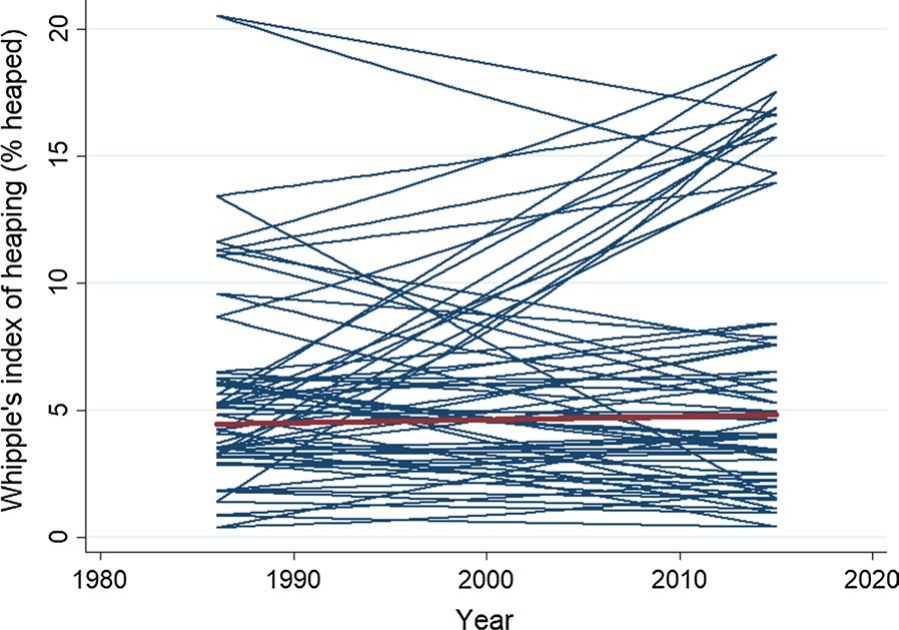
Estimated median predicted proportion of ages heaped by country across survey year

The introduction of the random slope parameter proved to significantly improve model fit based on a likelihood test. The predicted values by country from Model 2 are presented in Fig. 1. The overall trend in the proportion of age heaped in denoted by the red line within individual country trajectories denoted for each blue line. In general, there is a reasonable degree of clustering around the population line: the majority of countries have a portion of age heaped which is consistent over time, and in the range of between 2 and 6%.

Based on the predicted values of Whipple’s index of heaping, we identify countries with substantial differences between survey years 1987 and 2015 based on the residuals from model 2. We identify two countries with large predicted decreases in the proportion of age heaped, where we define a large decrease as being 4% points or more. Nigeria exhibits the largest decrease in the proportion of respondents reporting a heaped age, with a decline in the predicted value of Whipple’s index of 6.22% points, with the only other country exhibiting a large substantive decrease in the proportion of respondents with a heaped age being observed in Ghana with a fall of 4.28% points.

A number of countries exhibit substantive increases in the proportion of respondents reporting a heaped age, again defined as an increase of 4% points or more between the predicted values of Whipple’s index between 1987 and 2015. Sierra Leone, Chad and Ethopia demonstrate increases of 4.46% points, 7.38% points and 7.58% points respectively. We also note exceptionally large increases in the proportion of respondents with a heaped age in excess of 10% points between 1987 and 2015: Mali exhibits and increase of 11.78% points and Benin increases by 13.87% points.

### Conclusions

Data quality from retrospective sample surveys continues to be of major importance in social science, and basic demographic data is no exception. This paper therefore provides an assessment of the quality of age reported data within the DHS. We use all available DHS for the Sub Saharan Africa region to assess trends over time in the proportion of age reported which are heaped on terminal digits 0 and 5.

Out initial research hypothesis was that there may be a secular trend toward lower proportions of age heaped. However, in our analysis, we find no evidence of a significant decline in the proportion of ages heaped. That said the predicted probabilities are at a relatively low level for most countries, and are not a substantial concern. We do however identify some major outliers: Nigeria and Ghana have considerable falls in the proportion of ages heaped, while there have been dramatic increases in Sierra Leone, Ethiopia and Chad.

### Discussion

DHS data have provided detailed insight into developing countries but over time its methods have evolved. Research models can better cope with attitudes and behaviours in the field and the process, in recent years, allows for improved cultural translations. This has indeed reduced heaping in some areas and analyses from Sub-Saharan Africa show some improvement in data accuracy, along with increased levels of development. There appears to have been an adjustment for temporality being socially, culturally and economically defined, indicating that age heaping remains a mutable phenomenon.

This has been noted for other basic demographic information [8, 11, 12] where improvements in data collection procedures and provision of written information to increasingly literate populations [10] and better collection [8, 11, 12] techniques have been means of improving the accuracy of recalled data, for example birth weight [7, 8]. Potential explanations for improving data quality largely fall into the realms of better quality information being provided by respondents, and better collection techniques. Considering the effect of respondents, increasing utilisation of written demographic information made possible by greater levels of numeracy [10] has led to improvements over time in demographic data quality. Low numeracy and vague ideas about date of birth which were potentially down to low degrees of schooling [15]. Additionally, falling rates of malnutrition may be a potential explanation, as infant protein malnutrition syndrome was and is (in poorest economies) a limiting factor in an adult’s cognitive abilities (which can cause misreports in age) [16].

Consideration of the use of new techniques to reduce inaccuracies, such as calendars, as more recent versions of the DHS record additional variables Similar technique of alternate measures of *timepaths* using ‘local calendars’ that referenced local events and festivals which corresponded to the individual’s personal life [12]. This method is relatively successful in that respondents memory was triggered resulting in less duration heaping.

These advancements framed the motivation for our research hypothesis that the prevalence of age heaping would fall across time. While we find little evidence of this- there is no significant year effect in our models—indicating no movement toward secular improvement in the quality of age data. That said, out initial expectation of severe bias in certain contexts based on historic census information [2, 3] was also misplaced. While the lack of improvement in age data quality in the DHS is disappointing, this should be tempered by the fact the level of distortion is low to begin with. We do note some heterogeneity when taking country context into account, with some countries somewhat large changes in the degree of age misreporting. Tentatively, these changes can be explained by economic performance: relatively high growth rates in Nigeria and Ghana compared to moribund economic growth in Ethiopia and Chad exacerbated by internal conflict and violence which may have disrupted vital registration procedures. In any case, this study highlights the need to take into account country context when analysing data quality, even for standardised datasets such as the DHS.

## Limitations

- This analysis is only able to identify the proportion of ages in a population with digit preference, not whether individuals are misreporting their age.
- National level averages are produced: the likelihood of heaping is likely to vary between sub national groups e.g. better educated women are less likely to misreport their age than women with low educational attainment due to better numeracy [10].
